# Network pharmacology-based and clinically relevant prediction of the active ingredients and potential targets of Chinese herbs in metastatic breast cancer patients

**DOI:** 10.18632/oncotarget.15351

**Published:** 2017-02-15

**Authors:** Yu Mao, Jian Hao, Zi-Qi Jin, Yang-Yang Niu, Xue Yang, Dan Liu, Rui Cao, Xiong-Zhi Wu

**Affiliations:** ^1^ Tianjin Medical University Cancer Institute and Hospital, National Clinical Research Center for Cancer, Key Laboratory of Cancer Prevention and Therapy, Tianjin, 300060, China; ^2^ Tianjin Medical University, Tianjin, 300070, China; ^3^ Tianjin Children's Hospital, Tianjin, 300134, China; ^4^ Zhong-Shan-Men Inpatient Department, Tianjin Medical University Cancer Institute and Hospital, Tianjin, 300060, China

**Keywords:** metastatic breast cancer, Chinese herbal medicine, network pharmacology, estrogen receptor, HSP90

## Abstract

Chinese Herbal Medicine (CHM) plays a significant role in breast cancer treatment. We conduct the study to ascertain the relative molecular targets of effective Chinese herbs in treating stage IV breast cancer.

Survival benefit of CHM was verified by Kaplan-Meier method and Cox regression analysis. A bivariate correlation analysis was used to find and establish the effect of herbs in complex CHM formulas. A network pharmacological approach was adopted to explore the potential mechanisms of CHM.

Patients in the CHM group had a median survival time of 55 months, which was longer than the 23 months of patients in the non-CHM group. Cox regression analysis indicated that CHM was an independent protective factor. Correlation analysis showed that 10 herbs were strongly correlated with favorable survival outcomes (*P*<0.01). Bioinformatics analyses suggested that the 10 herbs might achieve anti-breast cancer activity primarily through inhibiting HSP90, ERα and TOP-II related pathways.

## INTRODUCTION

As the most general cancer in female, breast cancer (BC) is the second cause of cancer death among women all over the world, second only to lung cancer. [1–3]. It's estimated that breast cancer account for 15% of newly diagnosed cancers in Chinese women [4]. The mortality of breast cancer has decreased since the 1990s due to constant efforts in the process of screening, early stage diagnosis and systematic treatment [5]. Metastatic breast cancer (MBC) patients in the late stage of the disease have also benefited a lot from several lines of treatment. The overall survival time of MBC patients has been improved [6–8]. However, as we can see from a previous study, the 5-year survival rate of patients with MBC was only 24%, and the median survival time has not improved significantly in recent years [2, 8].

Chinese Herbal Medicine has been one of the most frequently used alternative treatments for different types of cancer. Both the single Chinese herb and the traditional Chinese herbal formulations have proven to be effective not only in reducing uncomfortable symptoms, such as pain, vomiting, diarrhea, fatigue and leucopenia after surgery and chemotherapy, but also in improving the survival benefits [9–11]. There are also a variety of single Chinese herbs that may be important components in Chinese formulations that have shown anti-proliferative and anti-migration activity in relevant experimental studies [12–15]. However, the specific survival benefits of CHM treatment on MBC patients remain unconfirmed and the mechanisms need to be further clarified.

**Figure 1:**
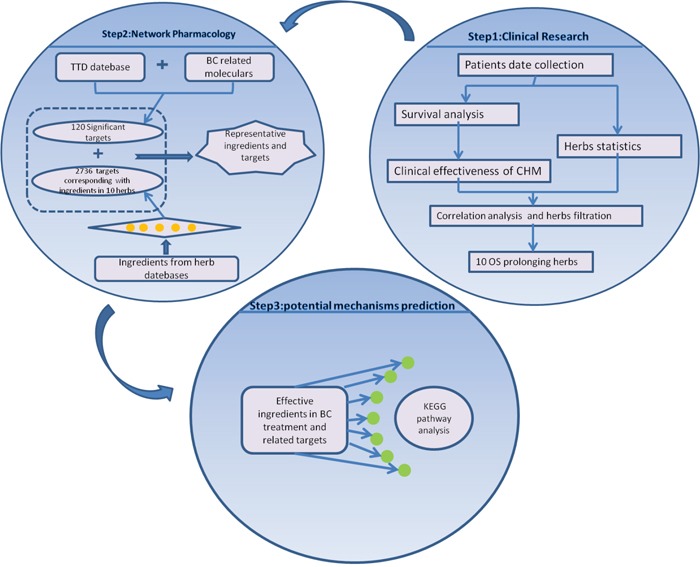
Process overview

Cancer is a disease in which multiple genes participate in a cumulative and gradual conversion of healthy cells into tumor cells. In addition, it's extremely difficult to analyze the complex compositions in herbal formulas merely through traditional experimental ways. Therefore, network pharmacology, which clarifies the potential mechanisms of complex ingredients through large data set analysis, is a suitable approach to meet this challenge and determine the synergistic effects in cancer treatment that incorporates holistic and systemic views [16, 17]. Thus, we intend to conduct the study in order to explore the potential mechanisms of CHM treatment on MBC using a systematic approach that integrates target prediction and network analysis based on clinical data, just as shown in Figure 1.

## RESULTS

### Survival characteristics

**Figure 2:**
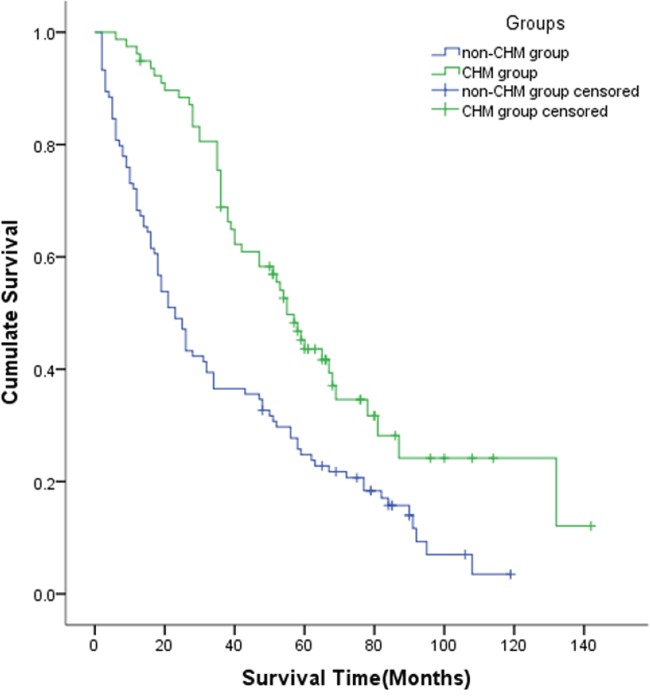
Kaplan-Meier Curve between the CHM and Non-CHM Groups Patients with CHM treatment had a longer median survival time than those without CHM treatment (55 months VS 23 months, *P* < 0.001). CHM: Chinese Herbal Medicine.

Overall 182 MBC patients were brought into the study. Among that 78 were CHM using patients and 104 under non-CHM treatment. The univariate analysis revealed that ER positive (*P* < 0.001), PR positive (*P* < 0.001), endocrine therapy (*P* = 0.015) and CHM treatment (*P* < 0.001) were protective factors. High blood CA-153 (*P* = 0.004) and CEA (*P* = 0.001) were linked with poorly reduced median overall survival, as shown in Table 1. Shown by the Cox regression analysis results, among the protective factors, CHM was an independent one. The hazard ratio (HR = Exp[β]) of CHM treatment was 0.591 and the associated 95% confidence intervals ranged from 0.395 to 0.885 (*P* < 0.05). The median survival time of CHM group (55 months) was longer than that of the non-CHM group (23 months). The 1-, 3- and 5-year survival rates for the CHM and non-CHM groups were 96.0%, 69.0%, 44.0%, and 72.1%, 36.5%, 24.7%, respectively (*P* < 0.001). Overall survival curves for CHM and non-CHM groups are shown in Figure 2. The baselines of the patient demographics were equal between patients with and without CHM treatment (Supplementary Table 1). The survival benefits among subgroups in CHM group differed (*P=0.023)*. Patients in Subgroup1 (ER+ and/or PR+, HER-2- ) benefit most from CHM. The median survival time is 67 months. Patients in Subgroup3(ER- and PR -, HER-2+) benefit least. The median survival time is only 38 months (Supplementary Figure 1, Supplementary Table 5).

**Table 1:** Univariate and multivariate analyses of variables influencing survival of 182 patients with MBC

Characteristics	N (%)	Univariate Analysis		Multivariate analysis		
		*P* Value	Β	Exp(β)	95% CI for Exp(β)	*P*
**Age(year)**		0.937	-	-	-	-
<50	57(31.3)					
≥50	125(68.7)					
**Pathological type**		***0.001***	-	-	-	-
Invasive ductal carcinoma	125(68.7)					
others	57(31.3)					
**The first metastasis place**		0.052	-	-	-	-
Bone	79(43.4)					
non-bone	103(56.6)					
**Metastatic style**		0.113	-	-	-	-
Single-position	83(45.6)					
Muti-position	99(54.4)					
**ER**		***<0.001***	−0.396	0.673	0.357-1.266	0.673
High	89(48.9)					
Normal	93(51.1)					
**PR**		***<0.001***	−0.272	0.788	0.455-1.275	0.301
High	72(39.6)					
Normal	110(60.4)					
**HER-2**		0.083	-	-	-	-
High	75(41.2)					
Normal	107(58.8)					
**CA-153**		***0.004***	0.444	1.559	0.965-2.519	***0.007***
High	50(27.5)					
Normal	89(48.9)					
Missing	43(23.6)					
**CEA**		***0.001***	0.431	1.539	0.948-2.497	0.081
High	51(28.0)					
Normal	90(49.5)					
Missing	41(22.5)					
**Surgery**		0.764	-	-	-	-
Yes	165(90.7)					
No	17(9.3)					
**Numbers of chemotherapy**		0.840	-	-	-	-
Less than 3 cycles	22(12.1)					
3 cycles and more	160(87.9)					
**Radiotherapy**		0.729	-	-	-	-
Yes	123(67.6)					
No	59(32.4)					
**Endocrine theraphy**		***0.015***	−0.238	1.516	0.440-1.412	0.424
Yes	90(49.5)					
No	92(50.5)					
**CHM**		***<0.001***	**-0.525**	**0.591**	**0.395-0.885**	***0.011***
Yes	78(42.9)					
No	104(57.1)					
**Target theraphy**		0.552		**-**	**-**	**-**
Yes	123(67.6)		**-**			
No	59(32.4)					

Abbreviations: CHM: Chinese Herbal Medicine; ER: Estrogen Receptor PR:Progesterone Receptor.

CA-153: Carbohydrate Antigen 153 CEA: Carcino-Embryonic Antigen.

### Identification of candidate protein targets associated with BC therapy

Although there are hundreds of significant genes and proteins that were abnormally expressed in BC cells, only a small number of them were identified as candidate BC targets. The Therapeutic Target Database (TTD) is a database for us to get information of therapeutic proteins and nucleic acid targets both known and explored. Therefore, we searched the TTD database and obtained 84 candidate BC targets. The other 36 candidate targets were from studies that were widely quoted. More detailed information of these BC targets was listed as Supplementary Table 2. Table 2 shows the online enrichment analysis results.

**Table 2:** Pathways associated with 120 candidate breast cancer targets according to the enrichment analysis based on KEGG and REACT pathway

Pathway	Annotated Targets Quantity	Annotated Genes	Corrected *P*-value	Pathway ID
Pathways in cancer	27	AKT1|BAX|BRCA2|CASP3|CASP9|CCND1|CDH1|CDK4|CDK6|EGFR|HDAC1|IGF1R|MAP2K1|MDM2|MMP1|MMP2|MMP9|mTOR|NFKB1|PDGFRA|PIK3CG|PIK3R1|PRKCG|RET|RUNX1|RXRB|TGFB1	1.22E-29	KEGG:05200
Cell Cycle	14	BRCA2|CCND1|CCND2|CCND3|CDC25A|CDK4|CDK6|CSNK2A1|DHFR|HDAC1|MDM2|TOP2A|TUBB|TYMS	6.97E-08	REACT:115566
Focal adhesion	13	AKT1|CCND1|CCND2|CCND3|EGFR|IGF1R|MAP2K1|PDGFRA|PIK3CG|PIK3R1|PRKCG|RARG|SRC	5.61E-12	KEGG:04510
ErbB signaling pathway	11	AKT1|CCND1|EGFR|ERBB3|MAP2K1|MTOR|NRG1|PIK3CG|PIK3R1|PRKCG|SRC	3.26E-13	KEGG:04012
MAPK signaling pathway	10	AKT1|CASP3|EGFR|HSPB1|MAP2K1|NFKB1|PDGFRA|PRKCG|TGFB1|TNF	1.18E-06	KEGG:04010
p53 signaling pathway	9	BAX|CASP3|CASP9|CCND1|CCND2|CCND3|CDK4|CDK6|MDM2	4.81E-11	KEGG:04115
Endocytosis	9	EGFR|ERBB3|IGF1R|MDM2|PDGFRA|RAB7A|RET|SRC|TGFB1	1.18E-08	KEGG:04144
Cytokine-cytokine receptor interaction	9	CCL2|CCL5|CXCL1|EGFR|PDGFRA|PRL|PRLR|TGFB1|TNF	9.18E-08	KEGG:04060
VEGF signaling pathway	8	AKT1|CASP9|HSPB1|MAP2K1|PIK3CG|PIK3R1|PRKCG|SRC	7.71E-11	KEGG:04370
Apoptosis	8	AKT1|BAX|CASP3|CASP9|NFKB1|PIK3CG|PIK3R1|TNF	1.66E-10	KEGG:04210
Jak-STAT signaling pathway	8	AKT1|CCND1|CCND2|CCND3|PIK3CG|PIK3R1|PRL|PRLR	1.54E-08	KEGG:04630
Chemokine signaling pathway	8	AKT1|CCL2|CCL5|CXCL1|MAP2K1|NFKB1|PIK3CG|PIK3R1	7.63E-08	KEGG:04062
Toll-like receptor signaling pathway	7	AKT1|CCL5|MAP2K1|NFKB1|PIK3CG|PIK3R1|TNF	2.56E-08	KEGG:04620
Steroid hormone biosynthesis	6	AKR1C1|CYP19A1|CYP1B1|HSD17B1|STS|SULT1E1	1.48E-08	KEGG:00140
Gap junction	6	EGFR|MAP2K1|PDGFRA|PRKCG|SRC|TUBB	3.10E-07	KEGG:04540
Tight junction	6	AKT1|CDK4|CDK6|CSNK2A1|PRKCG|SRC	3.09E-06	KEGG:04530
Natural killer cell mediated cytotoxicity	6	CASP3|MAP2K1|PIK3CG|PIK3R1|PRKCG|TNF	3.48E-06	KEGG:04650
Regulation of actin cytoskeleton	6	EGFR|MAP2K1|PDGFRA|PIK3CG|PIK3R1|RARG	4.01E-05	KEGG:04810
NOD-like receptor signaling pathway	5	CCL2|CCL5|CXCL1|NFKB1|TNF	8.77E-07	KEGG:04621
Adherens junction	5	CDH1|CSNK2A1|EGFR|IGF1R|SRC	2.76E-06	KEGG:04520
GnRH signaling pathway	5	EGFR|MAP2K1|MMP2|MMP9|SRC	1.49E-05	KEGG:04912
Cell-Cell communication	5	CDH1|CDH2|KRT5|PIK3R1|SRC	4.44E-05	REACT:111155
Insulin signaling pathway	5	AKT1|MAP2K1|MTOR|PIK3CG|PIK3R1	5.81E-05	KEGG:04910
mTOR signaling pathway	4	AKT1|MTOR|PIK3CG|PIK3R1	2.02E-05	KEGG:04150

### Candidate herbs associated with breast cancer

All the Chinese herbal formulas used by the 78 patients were collected; these formulas included a total of 231 types of herbs. Of those 231 herbs, 48 types of herbs, whose frequency of use was more than 10% of the total frequency of all herbs, were included for correlation analysis. Spearman bivariate correlate analysis showed that 20 herbs positively related to survival time and the correlation coefficient of 10 herbs was ≧ 0.350 (*P* < 0.01). The 10 herbs with high correlation coefficients were *Cervus Nippon Temminck* (NT), *Ginger Charcoal* (GC), *Citri Reticulatae Pericarpium Viride* (RP), *Phytolaccae Radix* (PR), *Licorice* (Lic), *Trichosanthes Kirilowii Maxim* (KM), *Citri Reticulatae Folium* (CR), *Panax Notoginseng* (PN), *Epimedium Herb* (EH), *Fritillariae Thunbergii Bulbus* (FTB).

### Target prediction through cI-cT network

Following the drug target prediction in the TCMSP and TCMID databases, we assembled the druggable proteins as putative targets for composite compounds contained in the 10 herbs (Supplementary Table 3). The potential ingredients predicted in the 10 herbs and their candidate breast cancer-related targets are shown in Supplementary Table 4. A total of 1192 composite ingredients were present in these 10 herbs and 254 of those ingredients have some effect against breast cancer.

**Figure 3:**
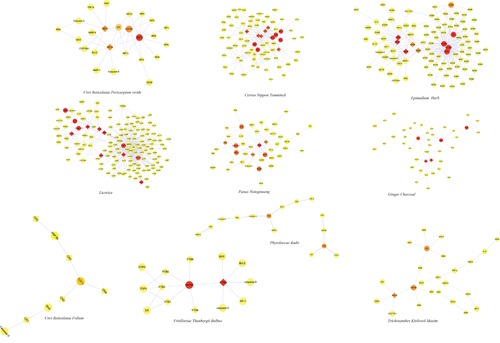
The Ingredient-target Networks The diamond nodes represent ingredients; the circular nodes represent targets; and the colors of the nodes are illustrated from red to yellow in descending order of degree values. (NT, *Cervus Nippon Temminck;* GC, *Ginger Charcoal;* RP, *Citri Reticulatae Pericarpium Viride;* PR, *Phytolaccae Radix;* Lic, *Licorice;* KM, *Trichosanthes Kirilowii Maxim;* CR, *Citri Reticulatae Folium;* PN, *Panax Notoginseng;* EH, *Epimedium Herb;* FTB, *Fritillariae Thunbergii Bulbus*).

In order to show the link between 10 herbs and BC related targets in an image model, the candidate ingredient-target network (cI-cT) was adopted. In this work, ingredients and targets were represented with nodes and mapped onto interlaced network. As produced in Figure 3, one target in the network may interact with one or more ingredients and vice versa. Thus, the relations between candidate ingredients and BC targets were vividly displayed. Based on the cI-cT network, the potential ingredients and putative major targets were generated (Table 3). HSP90, ERα and TOP-II were suppressed by multiple ingredients in the 10 herbs. Among this, HSP90 and ERα were suppressed by almost half of the ingredients. So one main anti-BC mechanisms might be HSP90 inhibitory activity. In addition, some proteins involving proliferation and apoptosis processes, such as EGFR, Src, HER2, caspase-3 and Bcl-2, could be hit by more than one ingredient. Inflammatory cytokines like IL-6, IL-1β and TNF-α were also suppressed by multiple ingredients.

**Table 3:** The major ingredients and major targets of 10 herbs

Chinese Name	Latin name	NO. of ingredients	Major ingredients	Number of targets	Major targets	Correlation coefficient	*P*-value
醋青皮	*Citri Reticulatae Pericarpium viride*	12	Naringenin (RP-07) Nobiletin (RP-10)	13	TOPII HSP90 ER	0.471	<0.001
醋商陆	*Phytolaccae Radix*	6	Esculetin (PR-02) Xanthomicrol (PR-06)	9	HSP90 Caspase-3	0.433	<0.001
鹿角霜	*Cervus Nippon Temminck*	20	beta-estradiol (NT-1) Adenosine triphosphate (NT-3) alpha-estradiol (NT-4) Ceramide (NT-5) Cerebroside (NT-6)	50	Caspase-3 EGFR IL1β IL-6 Src TNF-α	0.396	0.001
橘叶	*Citri Reticulatae Folium*	5	Hesperidin (CR-1)	4	HSP90 TOPII	0.395	0.001
瓜蒌	*Trichosanthes Kirilowii Maxim*	14	Tricin (KM-13)	11	HSP90 Src	0.394	0.002
姜炭	*Ginger Charcoal*	27	6-gingerol (GC-10) beta-sitosterol (GC-12)	14	HSP90 ER TOPII	0.376	0.003
浙贝母	*Fritillariae Thunbergii Bulbus*	6	beta-sitosterol (FTB-03)	9	HSP90 TOPII	0.375	0.002
淫羊藿	*EpimediumHerb*	68	Apigenin (EH-16) Emodin(EH-26) Kaempferol (EH-46) Luteolin (EH-49) Quercetin (EH-53) Anhydroicaritin (EH-14) Artonin U (EH-17)	30	HSP90 TOPII ESR2 ER VEGFR Caspase 3 MMP-1 TNF-α	0.370	0.003
三七	*Panax Notoginseng*	16	Quercetin (PN-15) beta-sitosterol (PN-02) Ginsenoside rh2 (PN-06) beta-elemene (PN-01)	27	Caspase-3 HSP90 IL-6 TNF-α Bcl-2	0.358	0.004
甘草	*Licorice*	110	Ursolic acid (Lic-104) Quercetin (Lic-97) Naringenin (Lic-86) licochalcone a (Lic-72) Kaempferol (Lic-66) Gancaonin A/B/G/H1-Methoxyphaseollidin (Lic-11)	33	HSP90 TOPII ESR2 ER VEGFR Caspase 3 Bcl-2	0.352	0.005

Overall, 25 composite ingredients matching the putative breast targets were in high degree distributions. More interestingly, most of the composite ingredients in the 10 herbs were flavonoids. The ingredients with the most potential were common in some of the 10 herbs. For example, beta-sitosterol is a common ingredient shared by *Panax Notoginseng* (PN)*, Fritillariae Thunbergii Bulbus* (FTB), and *Ginger Charcoal* (GC). Oleanolic acid is shared by *Epimedium herb* (EH)*, Panax Notoginseng* (PN)*, Phytolaccae Radix* (PR) and *Cervus Nippon Temminck* (NT). Palmitic acid is shared by *Panax Notoginseng* (PN)*, Trichosanthes Kirilowii Maxim* (KM) and *Ginger Charcoal* (GC). Quercetin is shared by *Licorice* (Lic)*, Epimedium herb* (EH) and *Panax Notoginseng* (PN).

### Integral mechanisms of anti-BC herbs

**Figure 4:**
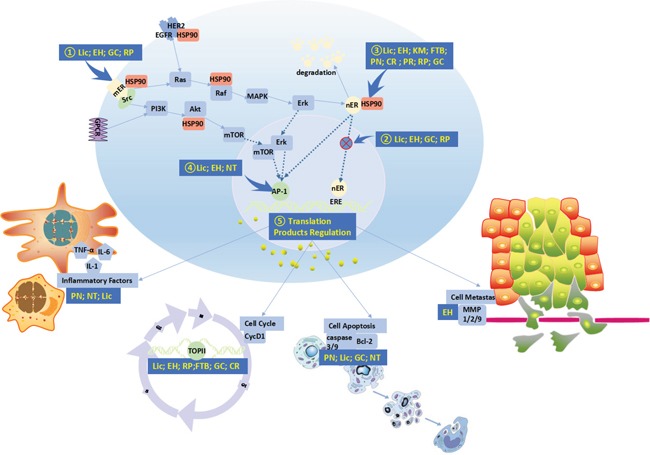
Associated pathways of the 10 Herbs Against Breast Cancer The 10 herbs might achieve anti-breast cancer activity primarily through the following routes: 1) Blockade of estrogen action via ER (estrogen receptor) antagonism. 2) Blocking the nuclear translocation of the ER. 3) Leading to degradation of the ER and oncoproteins through dissociation from HSP90. 4) Blocking transcriptional activity of AP-1 and ER. 5) Regulating the function of translation products by AP-1 and ER.

So as to clarify the primary pathways involved in the 10 herbs in BC treatment, we summarized the canonical pathways that were closely linked with BC among the potential targets, as shown in Supplementary Table 6. As a result, we identified 64 candidate targets for analysis. The 20 common candidate targets which were shared by more than 10 ingredients are listed in Table 4. HSP90, ERα and TOP-II are the top three targets. The putative targets were mapped to the most relevant pathways (Figure 4).

**Table 4:** Putative targets for main ingredients present in 10 Herbs

Putative Targets	NO. OfIngredients	The Top Drugs (NO. Of Ingredients)
HSP90	171	*Licorice(Lic,78), Epimedium Herb(EH,50)*
ER	142	*Licorice(Lic,89),Epimedium Herb(EH,25),Ginger Charcoal (GC, 13)*
TOPII	123	*Epimedium Herb(EH,52),Licorice(Lic,40),Citri Reticulatae Pericarpium viride(RP,10)*
VEGFR2	57	*Licorice(Lic,24),Epimedium Herb(EH,9),Radix Achyranthis Bidentatae(AB,2)*
Caspase-3	36	*Epimedium Herb(EH,32),Licorice(Lic,24)*,
AP-1	30	*Licorice(Lic,9),Epimedium Herb(EH,8),Cervus Nippon Temminck(NT, 6)*
Bcl-2	28	*Licorice(Lic,5), Panax notoginseng(PN,4)*
TNF-α	26	*Cervus Nippon Temminck(NT, 8),Epimedium Herb(EH,6),Panax notoginseng(PN,5)*
PIK3CG	20	*Licorice(Lic,18),Ginger Charcoal(GC, 2)*
BAX	19	*Licorice(Lic,4),Epimedium Herb(EH,4),Ginger Charcoal (GC,3)*
IL-6	19	*Licorice(Lic,4), Panax notoginseng(PN,4),Cervus Nippon,Temminck(NT,7)*
Caspase-9	18	*Panax notoginseng(PN,4),Licorice(Lic,3), Epimedium Herb(EH,3)*
MMP-9	17	*Cervus Nippon Temminck(NT,5),Epimedium Herb(EH,4)*
IL-1β	13	*Cervus Nippon Temminck(NT,8),Licorice(Lic,3)*
EGFR	13	*Cervus Nippon Temminck(NT,8)*
Src	13	*Cervus Nippon Temminck (NT,8),Trichosanthes Kirilowii Maxim (KM,3)*
MMP-1	12	*Epimedium Herb(EH,5), Licorice(Lic,3)*
MMP-2	11	*Licorice(Lic,3), Epimedium Herb(EH,2)*,
CCND1	10	*Epimedium Herb(EH,3), Licorice(Lic,3)*,
ErbB2	10	*Cervus Nippon Temminck (NT,3), Licorice(Lic,2), Panax notoginseng(PN,2)*

## DISCUSSION

Approximately 3-10% of BC cases were discovered to have distant metastasis at the initial diagnosis [32]. Stage IV MBC is still an incurable disease regardless of the progress in multidisciplinary therapy. The principal goal in treating MBC is to prolong the overall survival as long as possible. The median survival time of MBC is approximately 18-24 months, which is known from relative breast cancer statistics [2, 6, 8, 32, 33]. The survival benefits differ in regard to different therapeutic methods based on various molecular subtypes. HER-2+ MBC patients receiving trastuzumab plus chemotherapy benefited more than patients merely using chemotherapy [34]. For hormone receptor (HR) positive patients receiving endocrine treatment (ET), the benefit seems to be more promising [35]. In this study, the median survival of the control group was 23 months, which is consistent with the above studies. However, the improvement in overall survival is still limited. We indeed have not seen as many benefits as we expected in MBC, especially in adjuvant chemotherapy [36].

CHM is a significant and frequently used adjuvant therapy in China. The advantages of CHM in treating breast cancer are not only the effects in relieving uncomfortable symptoms such as pain, vomiting, leucopenia, and weakness but also in survival benefits [9, 10, 37]. Studies have shown encouraging results for breast cancer therapy [9, 38]. Our study has also revealed gratifying survival outcomes with an obvious improved median survival time of 55 months. Although the effects of CHM in breast cancer treatment have been explored in several clinical studies, the mechanisms in depth are not clear.

In our study, a total of 120 candidate targets in BC were obtained from the TTD database and the literature. The results of online enrichment analysis showed that these candidate BC targets were mostly belonged to intracellular signaling cascades in tumor growth and metastasis, such as the p53, ErbB, MAPK and VEGF signaling pathway (Supplementary Table 7).

The clinical findings demonstrated that 10 herbs were significantly associated with the survival benefits of MBC patients. A total of 1192 composite ingredients were present in these 10 herbs and 254 ingredients that have some effect against breast cancer were retained for further research. Among the 254 chemical components, 26 had a high degree of distribution and each of them hit the putative targets. Interestingly, most of the composite ingredients in the 10 herbs were flavonoids. Flavonoids have a wide range of properties and diverse biological effects. Numerous studies have reported interesting pre-clinical activity of flavonoids in kinds of cancers, suggesting their possible use in cancer prevention and treatment especially in BC [39–41]. Thus, it is of importance to probe the potential of these compounds in cancer treatments.

The ingredient-target herb networks showed that Chinese herbs played a role in treating BC through a biological network model. We identified major high-degree distribution ingredients in the 10 herbs. Our data showed that quercetin and kaempferol were shared by most of the 10 herbs and might play a major role in BC treatment.

Quercetin, a kind of flavonoids found in various plants, can inhibit the proliferation of a broad scope of malignancies [42–46]. It's also an ingredient in all of the 10 herbs. In BC cell lines, researches have reported that quercetin decreased the number of viable cells through increasing the level of apoptosis promoting protein Bax and decreasing the level the of Bcl-2 that inhibits cell apoptosis [47, 48]. The anti-tumor activity of kaempferol has been verified in BC. One study shows that kaempferol treatment arrest cell cycle in G2/M-phase and induces cell apoptosis [49]. Interestingly, quercetin and kaempferol are the main components of major flavonols in herbs and plant food and both also have antioxidant capacity [50].

As shown in Table 4, *Licorice* and *Epimedium Herb* played principal roles in the treatment of BC. *Licorice* is a kind of classic Chinese herb. It's commonly used in numerous formulas. *Licorice* contains various kinds of ingredients such as polysaccharides, triterpenoids and polyphenols. Studies have proved its pharmaceutical functions including anti-tumor effect. Glycyrrhizic acid can inhibit cyclooxygenase and lipoxygenase; suppress protein kinase C; reduce the expression of epidermal growth factor receptor (VEGFR). What's more, *Licorice* polyphenols has the effect of inducing apoptosis. [51, 52].

*Epimedium Herb* (EH) is a widely used Chinese medicine in numbers of formulas in treating bone diseases. Studies have shown that flavonoids of EH could reduce the bone loss [53]. Further studies proved that flavonoids in EH perform osteoblastic functions by stimulating ER. [54, 55]. The anti-cancer function of EH had not been widely reported, but the osteoblastic functions are rather attractive in treating BC bone metastases.

Among 64 major putative targets of the 10 herbs on BC therapy, HSP90, ERα and TOP-II are most directly depressed by these herbs. For BC therapies, the ER highly expressing in almost 70% of all breast tumors [56] is considered a molecule to target. As a member of the steroid receptor superfamily, ERα could promote various target cells to proliferate and differentiate. However, in the majority of breast cancers, ERα was greatly upregulated compared to normal breast cells and its expression was a hallmark of hormone-dependent tumor growth [57]. What's more, ERα play a significant role in BC initiation besides progression for benign breast epithelium with high ERα levels were more likely to become malignant ones [58]. Researchers have found that flavonoids can bind both isoforms of the ER, primarily as agonists competing with E2 [59], and induce biological responses traditionally associated with the binding of the natural hormone in a dose-dependent manner [60, 61]. As most of the chemical components with a high degree of distribution in the 10 herbs are flavonoids, it was not surprising that ERα was one of the putative targets.

HSP90 is a common kind of heat shock protein. It's of great help in maintaining the function of numerous intracellular proteins involving the process of apoptosis and cell cycle. [62]. When it comes to the BC cells, HSP90 plays an important role in keeping the stability of BC related proto-oncogenes such as estrogen receptor (ER), progesterone receptor (PR), Her2/neu and relative downstream proteins [63]. Over-expressed HSP90 was linked with bad prognosis in BC [64], indicating that HSP90 might be an effective target. The suppression of HSP90 may accelerate degradation of ER, PR, AKT, c-SRC, and RAF proteins.

Topoisomerase II alpha (TOP2A) is an important enzyme in the process of DNA replication. It has already become the target of some anticancer drugs. TOP2A is closely linked with the ERB2/HER-2/neu oncogene in the process of amplification. [65–66]. It would be interesting to explore the anti-cancer effect of the 10 herbs through DNA topoisomerase II-alpha-dependent cellular cytotoxicity.

In the present study, cancer-related targets involved in different stages of oncogenesis were included in the targets, determined through a network analysis, of 10 herbs. To better understand the functions of the targets associated with these 10 herbs, we analyzed the related pathways. As discussed before, most of the composite ingredients in the 10 herbs were flavonoids, and evidence supported the potential of these compounds to target different pathways and mechanisms underlying the complexity of cancer. These properties seem to be largely linked to their relatively simple structure: the presence of conjugated electron systems and aromatic rings make them stable and reactive; whereas, their overall structure allows them to act as substrates, inhibitors or agonists for numerous enzymes or molecules in cancer development and progression. Because of the important role of the ER in BC development, inhibition of the ER by flavonoids may be the most important mechanism in BC treatment. Therefore, by summarizing the canonical pathways that are highly correlated with BC and that are among the potential targets, the 10 herbs might achieve anti-breast cancer activity primarily through the following routes: 1) blockade of estrogen action via ER (estrogen receptor) antagonism; 2) blocking the nuclear translocation of the ER; 3) leading to degradation of the ER and oncoproteins through dissociation from HSP90; 4) blocking the transcriptional activity of AP-1 and the ER; 5) regulating the function of translation products by AP-1 and the ER.

Although network pharmacology is a promising method for identifying potential targets and active ingredients, there are some drawbacks that affect the analysis results. (i) The ingredients of the herbs were screened based on DL values, which might be inconsistent with the precise ingredients; (ii) the validated targets of these ingredients might be influenced by highly related studies; and (iii) the accuracy of target prediction depends on the target prediction tools.

## MATERIALS AND METHODS

### Patient characteristics

Patients with metastatic breast cancer between June 2004 and December 2011 in Tianjin Medical University Cancer Hospital were retrospectively studied. Information about the patients was collected from inpatient and outpatient medical records in Tianjin Medical University Cancer Hospital or from direct patient follow-up visits. After analysis of the data gathered and telephone follow ups, a total of 182 patients with MBC were available for this study.

The following is major inclusion criteria: aged over 18; at least one metastatic organ diagnosed by biopsy specimen obtained from puncture or imageological diagnosis at the first hospital visit or developed metastasis after therapy; received chemotherapy no less than three times; and patients in the CHM group received CHM treatment ≧ 4 weeks. Major exclusion criteria included the following: men; serious complications (liver failure, respiratory failure, severe mental disorder, severe cardiac diseases, etc.) concurrent cancer; incomplete medical records; without exact metastatic time; and failed to follow up.

### Treatment

MBC patients in the non-CHM group had accepted conventional western medicine (WM), including chemotherapy, radiotherapy and targeted therapy. Most of the ER positive patients also received endocrine therapy. All the patients in non-CHM group have never received any traditional Chinese medicine during and after hospitalization.

For the CHM group, the patients were treated by TCM formulas according to the different syndrome types in addition to conventional WM. In general, the formulas used in the present study contained 20-30 types of Chinese herbal substances. The patient formulas were collected every 2 weeks, because the formulas were modulated judged from symptoms changes in each 2 weeks. The formula collection work was started from the first CHM treatment time to the date the patients died or the time of data closure. At the end of the follow-up process, the CHM formulas of the 78 patients in the CHM group were gathered for us to analyze. The MBC patients had received CHM treatment for 22896 days altogether. To clearly distinguish the commonly used herbs, the single herb frequency/total frequency was used. The herbs with frequency >10% (single frequency/total frequency) were used to make bivariate correlation analysis. Herbs with correlation coefficient ≧ 0.350 (*P* < 0.01) were included for further network pharmacology dissection. Herbs with frequency <10% were not commonly used, and the use of which was to relieve various uncomfortable symptoms and they were changed after a 14-day interval.

### Candidate BC-related targets

Candidate BC targets were obtained from two existing resources: (1) Therapeutic Target Database [18] (http://bidd.nus.edu.sg/group/cjttd/ttd_home.asp, Version 4.3.02 release on Sep 15th, 2013). Therapeutic protein targets, both known and explored, were used as BC targets. Totally, we got 84 candidate BC-related targets in the database. (2) Literature, a total of 36 BC targets were from studies that are widely quoted [19–21]. Pathway enrichment analysis for candidate BC targets were performed online using the genes of candidate BC targets [22] (http://ctdbase.org/tools/analyzer.go).

### Herb formulation ingredient collection and target fishing

As in our previous study [23], the chemical ingredients were collected from the Traditional Chinese Medicine Systems Pharmacology (TCMSP) Database [24] (http://lsp.nwu.edu.cn/), and Traditional Chinese Medicine Integrative (TCMID) Database (http://www.megabionet.org/tcmid/) [25] and subsequently screened according to drug-likeness (DL) values, considering both pharmaco-dynamic and pharmaco-kinetic properties. The ingredients with a DL value higher than 0.18 were retained for further investigation.

Target fishing was conducted to confirm or predict the potential targets of the chemical ingredients. The validated targets were extracted from the Herbal Ingredients’ Targets (HIT) Database [26] (http://lifecenter.sgst.cn/hit/). The predicted targets were obtained using ChemMapper [27] (http://lilab.ecust.edu.cn/chemmapper/), an online tool for predicting targets based on 3D similarity.

### Network construction and analysis

Ten herbs (*Cervus Nippon Temminck* (NT), *Ginger Charcoal* (GC), *Citri Reticulatae Pericarpium Viride* (RP), *Phytolaccae Radix* (PR), *Licorice* (Lic), *Trichosanthes Kirilowii Maxim* (KM), *Citri Reticulatae Folium* (CR), *Panax Notoginseng* (PN), *Epimedium Herb* (EH), *Fritillariae Thunbergii Bulbus* (FTB)) were found to have high correlation coefficients. The ingredients-targets networks were constructed for these herbs using Cytoscape software [28] (Version 3.2.1). The network was analyzed using Cytoscape software with the CentiScaPe plugin [29] to calculate topological parameters, including the degree, betweenness, closeness and centroid. The significant nodes representing putative major ingredients and major targets of herbs were explored.

### Ethics approval and consent to participate

This study was performed with the approval of the Ethics Committee of Tianjin Medical University. Exemption from obtaining informed consent was approved through the Ethics Committee as this study was retrospective and many patients had died prior to conducting the study. This study conformed to the standards of the Declaration of Helsinki. The informed consent was not required because personal identifying information was not involved.

### Statistical analysis

Overall survival was defined as the time from the day MBC was diagnosed to the day the patient died of breast cancer or the last day the patient was followed. Baseline comparison was analyzed by the χ^2^ test. Kaplan-Meier curves and Multivariate Cox regression analysis were used to evaluate the differences in survival time. *P* < 0.05 was considered statistically significant. Spearman bivariate correlation analysis was used to determine the correlation between herbs and survival time. *P* < 0.01 was considered statistically significant. Statistical analyses were performed by using SPSS 19.0.

## SUPPLEMENTARY FIGURE AND TABLES













## Abbreviations

BC: breast cancer
Bcl-2: B-cell lymphoma-2
CA-153: carbohydrate antigen-153
CDK1: cyclin-dependent kinases1
CEA: carcino-embryonic antigen
CHM: Chinese Herbal Medicine
cI-cT: candidate ingredient-target
CR: Citri Reticulatae Folium
DL: drug-likeness
DNA: deoxyribonucleic acid
EGFR: epidermal growth factor receptor
EH: Epimedium Herb
ER: estrogen receptor
PR: progesterone receptor
ERE: estrogen response element
ET: endocrine treatment
FTB: Fritillariae Thunbergii Bulbus
GC: Ginger Charcoal
Her2: human epidermalgrowth factor receptor-2
HIT: Herbal Ingredients’ Targets
HSP90: heat shock protein90
HR: hazard ratio
IL-6: interleukin- 6
IL-1β: interleukin- 1β
KM: Trichosanthes Kirilowii Maxim
Lic: Licorice
MAPK: mitogen-activated protein kinase
MBC: Metastatic breast cancer
NT: Cervus Nippon Temminck
OS: Overall Survival
PN: Panax Notoginseng
PR: Phytolaccae Radix
RP: Citri Reticulatae Pericarpium Viride
Scr: Sarcoma
TCMID: Traditional Chinese Medicine Integrative Database
TCMSP: Traditional Chinese Medicine Systems Pharmacology
TNF-α: Tumor Necrosis Factor
TOP-II: Topoisomerase II
TTD: Therapeutic Target Database
VEGF: vascular endothelial growth factor
VEGFR: vascular endothelial growth factor receptor
WM: western medicine
AP-1: activator protein 1
