# Supplementary material for: Network pharmacology-based and clinically relevant prediction of the active ingredients and potential targets of Chinese herbs in metastatic breast cancer patients
Source: Oncotarget. 2017 Feb 15;8(16):27007–21. doi: 10.18632/oncotarget.15351 (PMC5432314; doi:10.18632/oncotarget.15351)
Supplement: Supplementary file 1 [file oncotarget-08-27007-s001.pdf]

Network pharmacology-based and clinically relevant prediction of the active ingredients and potential targets of Chinese herbs in metastatic breast cancer patients

SUPPLEMENTARY FIGURE AND TABLES

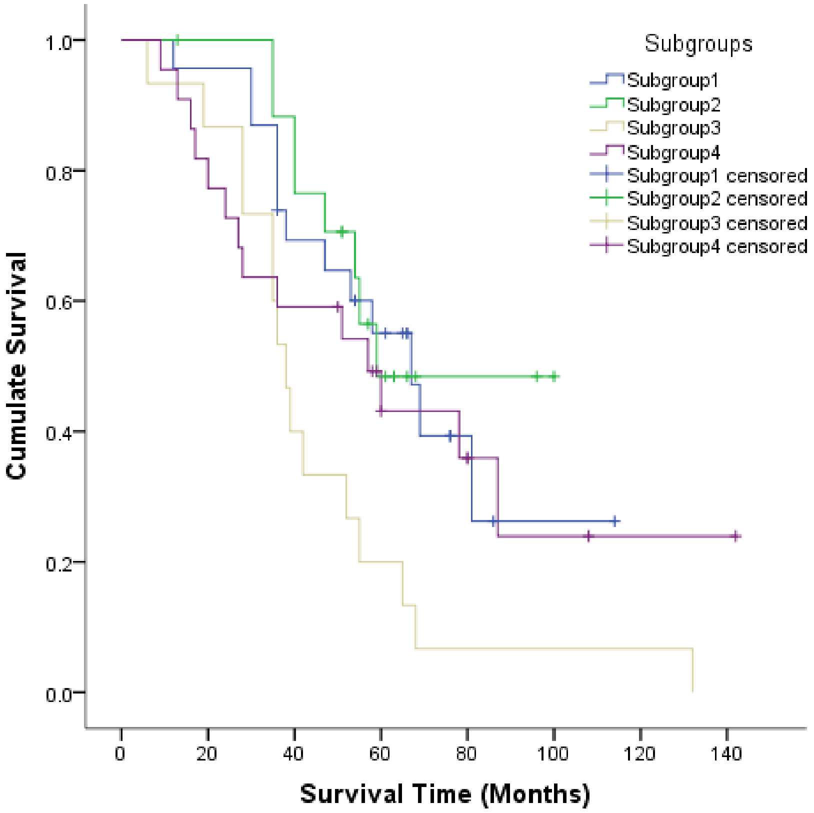

Supplementary Figure 1: Kaplan-Meier Curve between subgroups in CHM group. Patients in Subgroup1 benefit most from CHM whose median survival time is 67 months. Patients in Subgroup3(ER and PR negative but HER-2 positive) benefit least. The median survival time is only 38 months.

Subgroups1: ER+ and/or PR +, HER-2 -
Subgroups2: ER+ and/or PR +, HER-2 +
Subgroups3: ER-, PR -, HER-2 +
Subgroups4: ER-, PR -, HER-2 -

Supplementary Table 1: Details of baseline characteristics of patients with stage IV breast cancer

Variable	CHM group N=78	Non-CHM group N=104	*P* value
**Age(year)**			0.645
<50/≥50	23/55	34/70	
**Pathological type**			0.072
Invasive ductal carcinoma/others	48/30	77/27	
**The first metastasis place**			0.966
Bone/non-bone	34/44	45/59	
**Metastatic style**			0.169
Single-position/Muti-position	31/47	52/52	
**ER**			0.392
Positive/Negative	41/ 37	48/ 56	
**PR**			0.965
Positive/Negative	31/47	41/63	
**Her-2**			0.794
Positive/Negative	33/ 45	42 /62	
**Surgery**			0.883
Yes/No	71/7	94/10	
**Numbers of chemotherapy**			0.115
< 3 cycles/≥3 cycles	6/72	16/88	
**Radiotherapy**			0.819
Yes/No	52/26	71/33	
**Target theraphy**			0.390
Yes/No	6/72	12/92	
Endocrine theraphy			0.304
Yes/No	42/36	48/56	

Abbreviations: CHM: Chinese Herbal Medicine; ER: Estrogen Receptor PR:Progesterone Receptor.

Supplementary Table 2: Candidate Protein Targets Associated with Breast Cancer Therapy

See Supplementary File 1

Supplementary Table 3: The Predicted/Validated Target Names of Each Ingredient

See Supplementary File 2

Supplementary Table 4: Potential Ingredients Predicted in the 10 Herbs and Their Candidate Breast Cancer-Related Targets

See Supplementary File 3

Supplementary Table 5: The different receptor subgroups survival beneficial in CHM group patients

Subroup Name	Subgroup Details	N (%)	Median Survival Time(Months)	3-year survival rate (%)	5-year survival rate (%)
Subroup1	ER+ and/or PR + HER-2 –	23(30)	67	73.9	55.0
Subroup2	ER+ and/or PR + HER-2 +	18(23)	59	88.2	48.4
Subroup3	ER- PR - HER-2 +	15(19)	38	53.3	20.0
Subroup4	ER- PR - HER-2 -	22(28)	57	59.1	43.1

Supplementary Table 6: GO analysis of CHM on breast cancer

See Supplementary File 4

Supplementary Table 7: Pathway analysis of breast cancer related targets

See Supplementary File 5
