# Supplementary material for: Network pharmacology-based and clinically relevant prediction of the active ingredients and potential targets of Chinese herbs in metastatic breast cancer patients
Source: Oncotarget. 2017 Feb 15;8(16):27007–21. doi: 10.18632/oncotarget.15351 (PMC5432314; doi:10.18632/oncotarget.15351)
Supplement: Supplementary file 5 [file oncotarget-08-27007-s005.docx]

| **Pathway** | **Pathway ID** | **P-value** | **Corrected P-value** | **Annotated Genes Quantity** | **Annotated Genes** | **Genome Frequency** |
| --- | --- | --- | --- | --- | --- | --- |
| Signal Transduction | REACT:111102 | 9.33E-19 | 1.17E-16 | 23 | AKR1C1\|AKT1\|BAD\|CASP3\|CASP9\|CCL2\|CCND1\|EGF\|EGFR\|ERBB2\|ERBB3\|HSPB1\|IGF1R\|MAPK1\|MMP3\|MMP9\|NFKB1\|PARP1\|PIK3CG\|SRC\|STAT1\|TGFB1\|VEGFA | 1918/41530 genes: 4.62% |
| Pathways in cancer | KEGG:05200 | 4.76E-34 | 5.95E-32 | 22 | AKT1\|BAD\|BAX\|BCL2\|CASP3\|CASP9\|CCND1\|CDK4\|CDK6\|EGF\|EGFR\|ERBB2\|IGF1R\|JUN\|MAPK1\|MMP2\|MMP9\|NFKB1\|PIK3CG\|STAT1\|TGFB1\|VEGFA | 333/41530 genes: 0.80% |
| Pancreatic cancer | KEGG:05212 | 3.62E-31 | 4.53E-29 | 15 | AKT1\|BAD\|CASP9\|CCND1\|CDK4\|CDK6\|EGF\|EGFR\|ERBB2\|MAPK1\|NFKB1\|PIK3CG\|STAT1\|TGFB1\|VEGFA | 70/41530 genes: 0.17% |
| Tuberculosis | KEGG:05152 | 2.21E-22 | 2.76E-20 | 14 | AKT1\|BAD\|BAX\|BCL2\|CASP3\|CASP9\|IL10\|IL1B\|MAPK1\|NFKB1\|SRC\|STAT1\|TGFB1\|TNF | 185/41530 genes: 0.45% |
| Focal adhesion | KEGG:04510 | 1.45E-21 | 1.82E-19 | 14 | AKT1\|BAD\|BCL2\|CCND1\|CCND2\|EGF\|EGFR\|ERBB2\|IGF1R\|JUN\|MAPK1\|PIK3CG\|SRC\|VEGFA | 211/41530 genes: 0.51% |
| Immune System | REACT:6900 | 1.96E-11 | 2.45E-09 | 14 | AKT1\|BAD\|BCL2\|CASP9\|EGF\|EGFR\|ERBB2\|ERBB3\|IL1B\|JUN\|MAPK1\|NFKB1\|SRC\|STAT1 | 1144/41530 genes: 2.75% |
| Disease | REACT:116125 | 7.13E-10 | 8.92E-08 | 13 | AKR1C1\|AKT1\|BAD\|CASP9\|EGF\|EGFR\|ERBB2\|ERBB3\|MAPK1\|PARP1\|SRC\|STAT1\|TGFB1 | 1232/41530 genes: 2.97% |
| Prostate cancer | KEGG:05215 | 3.25E-22 | 4.06E-20 | 12 | AKT1\|BAD\|BCL2\|CASP9\|CCND1\|EGF\|EGFR\|ERBB2\|IGF1R\|MAPK1\|NFKB1\|PIK3CG | 92/41530 genes: 0.22% |
| Toxoplasmosis | KEGG:05145 | 4.39E-20 | 5.48E-18 | 12 | AKT1\|BAD\|BCL2\|CASP3\|CASP9\|IL10\|MAPK1\|NFKB1\|PIK3CG\|STAT1\|TGFB1\|TNF | 136/41530 genes: 0.33% |
| Non-small cell lung cancer | KEGG:05223 | 1.02E-22 | 1.27E-20 | 11 | AKT1\|BAD\|CASP9\|CCND1\|CDK4\|CDK6\|EGF\|EGFR\|ERBB2\|MAPK1\|PIK3CG | 54/41530 genes: 0.13% |
| Colorectal cancer | KEGG:05210 | 7.83E-22 | 9.79E-20 | 11 | AKT1\|BAD\|BAX\|BCL2\|CASP3\|CASP9\|CCND1\|JUN\|MAPK1\|PIK3CG\|TGFB1 | 64/41530 genes: 0.15% |
| ErbB signaling pathway | KEGG:04012 | 5.54E-20 | 6.92E-18 | 11 | AKT1\|BAD\|CCND1\|EGF\|EGFR\|ERBB2\|ERBB3\|JUN\|MAPK1\|PIK3CG\|SRC | 92/41530 genes: 0.22% |
| MAPK signaling pathway | KEGG:04010 | 1.83E-14 | 2.29E-12 | 11 | AKT1\|CASP3\|EGF\|EGFR\|HSPB1\|IL1B\|JUN\|MAPK1\|NFKB1\|TGFB1\|TNF | 285/41530 genes: 0.69% |
| Melanoma | KEGG:05218 | 5.42E-19 | 6.77E-17 | 10 | AKT1\|BAD\|CCND1\|CDK4\|CDK6\|EGF\|EGFR\|IGF1R\|MAPK1\|PIK3CG | 71/41530 genes: 0.17% |
| Apoptosis | KEGG:04210 | 6.61E-18 | 8.26E-16 | 10 | AKT1\|BAD\|BAX\|BCL2\|CASP3\|CASP9\|IL1B\|NFKB1\|PIK3CG\|TNF | 90/41530 genes: 0.22% |
| Chagas disease (American trypanosomiasis) | KEGG:05142 | 3.29E-17 | 4.11E-15 | 10 | AKT1\|CCL2\|IL10\|IL1B\|JUN\|MAPK1\|NFKB1\|PIK3CG\|TGFB1\|TNF | 105/41530 genes: 0.25% |
| Hepatitis C | KEGG:05160 | 5.91E-16 | 7.39E-14 | 10 | AKT1\|BAD\|CCND1\|EGF\|EGFR\|MAPK1\|NFKB1\|PIK3CG\|STAT1\|TNF | 139/41530 genes: 0.33% |
| Bladder cancer | KEGG:05219 | 5.84E-19 | 7.30E-17 | 9 | CCND1\|CDK4\|EGF\|EGFR\|ERBB2\|MAPK1\|MMP2\|MMP9\|VEGFA | 42/41530 genes: 0.10% |
| Endometrial cancer | KEGG:05213 | 8.24E-18 | 1.03E-15 | 9 | AKT1\|BAD\|CASP9\|CCND1\|EGF\|EGFR\|ERBB2\|MAPK1\|PIK3CG | 55/41530 genes: 0.13% |
| Glioma | KEGG:05214 | 4.11E-17 | 5.14E-15 | 9 | AKT1\|CCND1\|CDK4\|CDK6\|EGF\|EGFR\|IGF1R\|MAPK1\|PIK3CG | 65/41530 genes: 0.16% |
| Chronic myeloid leukemia | KEGG:05220 | 1.60E-16 | 2.00E-14 | 9 | AKT1\|BAD\|CCND1\|CDK4\|CDK6\|MAPK1\|NFKB1\|PIK3CG\|TGFB1 | 75/41530 genes: 0.18% |
| Osteoclast differentiation | KEGG:04380 | 3.09E-14 | 3.86E-12 | 9 | AKT1\|IL1B\|JUN\|MAPK1\|NFKB1\|PIK3CG\|STAT1\|TGFB1\|TNF | 132/41530 genes: 0.32% |
| Measles | KEGG:05162 | 5.29E-14 | 6.62E-12 | 9 | AKT1\|CCND1\|CCND2\|CDK4\|CDK6\|IL1B\|NFKB1\|PIK3CG\|STAT1 | 140/41530 genes: 0.34% |
| Leishmaniasis | KEGG:05140 | 2.04E-14 | 2.56E-12 | 8 | IL10\|IL1B\|JUN\|MAPK1\|NFKB1\|STAT1\|TGFB1\|TNF | 74/41530 genes: 0.18% |
| VEGF signaling pathway | KEGG:04370 | 3.91E-14 | 4.89E-12 | 8 | AKT1\|BAD\|CASP9\|HSPB1\|MAPK1\|PIK3CG\|SRC\|VEGFA | 80/41530 genes: 0.19% |
| Small cell lung cancer | KEGG:05222 | 7.84E-14 | 9.80E-12 | 8 | AKT1\|BCL2\|CASP9\|CCND1\|CDK4\|CDK6\|NFKB1\|PIK3CG | 87/41530 genes: 0.21% |
| Amoebiasis | KEGG:05146 | 5.01E-13 | 6.26E-11 | 8 | CASP3\|HSPB1\|IL10\|IL1B\|NFKB1\|PIK3CG\|TGFB1\|TNF | 109/41530 genes: 0.26% |
| Toll-like receptor signaling pathway | KEGG:04620 | 5.01E-13 | 6.26E-11 | 8 | AKT1\|IL1B\|JUN\|MAPK1\|NFKB1\|PIK3CG\|STAT1\|TNF | 109/41530 genes: 0.26% |
| T cell receptor signaling pathway | KEGG:04660 | 5.39E-13 | 6.74E-11 | 8 | AKT1\|CDK4\|IL10\|JUN\|MAPK1\|NFKB1\|PIK3CG\|TNF | 110/41530 genes: 0.26% |
| Neurotrophin signaling pathway | KEGG:04722 | 2.24E-12 | 2.80E-10 | 8 | AKT1\|BAD\|BAX\|BCL2\|JUN\|MAPK1\|NFKB1\|PIK3CG | 131/41530 genes: 0.32% |
| Cytokine-cytokine receptor interaction | KEGG:04060 | 9.14E-10 | 1.14E-07 | 8 | CCL2\|EGF\|EGFR\|IL10\|IL1B\|TGFB1\|TNF\|VEGFA | 278/41530 genes: 0.67% |
| p53 signaling pathway | KEGG:04115 | 1.52E-12 | 1.90E-10 | 7 | BAX\|CASP3\|CASP9\|CCND1\|CCND2\|CDK4\|CDK6 | 69/41530 genes: 0.17% |
| Rheumatoid arthritis | KEGG:05323 | 1.31E-11 | 1.64E-09 | 7 | CCL2\|IL1B\|JUN\|MMP3\|TGFB1\|TNF\|VEGFA | 93/41530 genes: 0.22% |
| Apoptosis | REACT:578 | 5.34E-10 | 6.68E-08 | 7 | AKT1\|BAD\|BAX\|BCL2\|CASP3\|CASP9\|TNF | 157/41530 genes: 0.38% |
| Developmental Biology | REACT:111045 | 1.04E-06 | 1.30E-04 | 7 | AKT1\|EGFR\|ERBB2\|MAPK1\|MMP2\|MMP9\|SRC | 475/41530 genes: 1.14% |
| Hemostasis | REACT:604 | 1.36E-06 | 1.71E-04 | 7 | AKT1\|EGF\|MAPK1\|PIK3CG\|SRC\|TGFB1\|VEGFA | 495/41530 genes: 1.19% |
| Amyotrophic lateral sclerosis (ALS) | KEGG:05014 | 4.69E-11 | 5.86E-09 | 6 | BAD\|BAX\|BCL2\|CASP3\|CASP9\|TNF | 56/41530 genes: 0.13% |
| Acute myeloid leukemia | KEGG:05221 | 5.24E-11 | 6.55E-09 | 6 | AKT1\|BAD\|CCND1\|MAPK1\|NFKB1\|PIK3CG | 57/41530 genes: 0.14% |
| Renal cell carcinoma | KEGG:05211 | 2.42E-10 | 3.03E-08 | 6 | AKT1\|JUN\|MAPK1\|PIK3CG\|TGFB1\|VEGFA | 73/41530 genes: 0.18% |
| GnRH signaling pathway | KEGG:04912 | 3.12E-09 | 3.90E-07 | 6 | EGFR\|JUN\|MAPK1\|MMP2\|MMP9\|SRC | 111/41530 genes: 0.27% |
| Jak-STAT signaling pathway | KEGG:04630 | 2.50E-08 | 3.13E-06 | 6 | AKT1\|CCND1\|CCND2\|IL10\|PIK3CG\|STAT1 | 157/41530 genes: 0.38% |
| Alzheimer's disease | KEGG:05010 | 4.46E-08 | 5.58E-06 | 6 | BAD\|CASP3\|CASP9\|IL1B\|MAPK1\|TNF | 173/41530 genes: 0.42% |
| Chemokine signaling pathway | KEGG:04062 | 8.80E-08 | 1.10E-05 | 6 | AKT1\|CCL2\|MAPK1\|NFKB1\|PIK3CG\|STAT1 | 194/41530 genes: 0.47% |
| Endocytosis | KEGG:04144 | 1.75E-07 | 2.19E-05 | 6 | EGF\|EGFR\|ERBB3\|IGF1R\|SRC\|TGFB1 | 218/41530 genes: 0.52% |
| Cellular responses to stress | REACT:120956 | 4.60E-07 | 5.75E-05 | 6 | CDK4\|CDK6\|JUN\|MAPK1\|NFKB1\|VEGFA | 257/41530 genes: 0.62% |
| Malaria | KEGG:05144 | 3.72E-09 | 4.65E-07 | 5 | CCL2\|IL10\|IL1B\|TGFB1\|TNF | 52/41530 genes: 0.13% |
| NOD-like receptor signaling pathway | KEGG:04621 | 8.46E-09 | 1.06E-06 | 5 | CCL2\|IL1B\|MAPK1\|NFKB1\|TNF | 61/41530 genes: 0.15% |
| Epithelial cell signaling in Helicobacter pylori infection | KEGG:05120 | 1.47E-08 | 1.84E-06 | 5 | CASP3\|EGFR\|JUN\|NFKB1\|SRC | 68/41530 genes: 0.16% |
| Adherens junction | KEGG:04520 | 2.96E-08 | 3.70E-06 | 5 | EGFR\|ERBB2\|IGF1R\|MAPK1\|SRC | 78/41530 genes: 0.19% |
| B cell receptor signaling pathway | KEGG:04662 | 3.16E-08 | 3.94E-06 | 5 | AKT1\|JUN\|MAPK1\|NFKB1\|PIK3CG | 79/41530 genes: 0.19% |
| Cell cycle | KEGG:04110 | 3.42E-07 | 4.27E-05 | 5 | CCND1\|CCND2\|CDK4\|CDK6\|TGFB1 | 127/41530 genes: 0.31% |
| Extracellular matrix organization | REACT:118779 | 1.28E-05 | 0.0016 | 5 | CASP3\|MMP2\|MMP3\|MMP9\|TGFB1 | 266/41530 genes: 0.64% |
| mTOR signaling pathway | KEGG:04150 | 5.84E-07 | 7.30E-05 | 4 | AKT1\|MAPK1\|PIK3CG\|VEGFA | 58/41530 genes: 0.14% |
| Steroid hormone biosynthesis | KEGG:00140 | 6.70E-07 | 8.38E-05 | 4 | AKR1C1\|CYP19A1\|CYP1B1\|SULT1E1 | 60/41530 genes: 0.14% |
| Fc epsilon RI signaling pathway | KEGG:04664 | 2.73E-06 | 3.41E-04 | 4 | AKT1\|MAPK1\|PIK3CG\|TNF | 85/41530 genes: 0.20% |
| Progesterone-mediated oocyte maturation | KEGG:04914 | 3.58E-06 | 4.47E-04 | 4 | AKT1\|IGF1R\|MAPK1\|PIK3CG | 91/41530 genes: 0.22% |
| Gap junction | KEGG:04540 | 4.81E-06 | 6.01E-04 | 4 | EGF\|EGFR\|MAPK1\|SRC | 98/41530 genes: 0.24% |
| Natural killer cell mediated cytotoxicity | KEGG:04650 | 2.32E-05 | 0.00291 | 4 | CASP3\|MAPK1\|PIK3CG\|TNF | 146/41530 genes: 0.35% |
| Insulin signaling pathway | KEGG:04910 | 2.45E-05 | 0.00306 | 4 | AKT1\|BAD\|MAPK1\|PIK3CG | 148/41530 genes: 0.36% |
| African trypanosomiasis | KEGG:05143 | 9.45E-06 | 0.00118 | 3 | IL10\|IL1B\|TNF | 36/41530 genes: 0.09% |
| Prion diseases | KEGG:05020 | 1.11E-05 | 0.00139 | 3 | BAX\|IL1B\|MAPK1 | 38/41530 genes: 0.09% |
| Innate Immune System | REACT:147795 | 2.13E-05 | 0.00266 | 3 | JUN\|MAPK1\|NFKB1 | 47/41530 genes: 0.11% |
| Type II diabetes mellitus | KEGG:04930 | 2.27E-05 | 0.00283 | 3 | MAPK1\|PIK3CG\|TNF | 48/41530 genes: 0.12% |
| TRAF6 Mediated Induction of proinflammatory cytokines | REACT:6782 | 4.44E-05 | 0.00555 | 3 | JUN\|MAPK1\|NFKB1 | 60/41530 genes: 0.14% |
| Adipocytokine signaling pathway | KEGG:04920 | 7.05E-05 | 0.00881 | 3 | AKT1\|NFKB1\|TNF | 70/41530 genes: 0.17% |
| Viral myocarditis | KEGG:05416 | 7.99E-05 | 0.00999 | 3 | CASP3\|CASP9\|CCND1 | 73/41530 genes: 0.18% |

**Table S6. GO analysis of CHM on breast cancer**
