# Supplementary material for: Network pharmacology-based and clinically relevant prediction of the active ingredients and potential targets of Chinese herbs in metastatic breast cancer patients
Source: Oncotarget. 2017 Feb 15;8(16):27007–21. doi: 10.18632/oncotarget.15351 (PMC5432314; doi:10.18632/oncotarget.15351)
Supplement: Supplementary file 6 [file oncotarget-08-27007-s006.docx]

| **Pathway** | **Annotated Genes Quantity** | **Annotated Genes** | **Pathway ID** | **P-value** |
| --- | --- | --- | --- | --- |
| Signal Transduction | 37 | AKR1C1\|AKT1\|CASP3\|CASP9\|CCL2\|CCL5\|CCND1\|CDK9\|CSNK2A1\|CXCL1\|EGFR\|ERBB3\|ESR1\|FNTA\|GRB7\|HDAC1\|HSD17B1\|HSPB1\|IGF1R\|LHCGR\|MAP2K1\|MDM2\|MMP9\|MTOR\|NFKB1\|NRG1\|PDGFRA\|PIK3CG\|PIK3R1\|PRKCG\|PRL\|PRLR\|SCUBE2\|SMAD9\|SRC\|TGFB1\|VIPR1 | REACT:  111102 | 1.89E-21 |
| Pathways in cancer | 27 | AKT1\|BAX\|BRCA2\|CASP3\|CASP9\|CCND1\|CDH1\|CDK4\|CDK6\|EGFR\|HDAC1\|IGF1R\|MAP2K1\|MDM2\|MMP1\|MMP2\|MMP9\|MTOR\|NFKB1\|PDGFRA\|PIK3CG\|PIK3R1\|PRKCG\|RET\|RUNX1\|RXRB\|TGFB1 | KEGG:05200 | 8.01E-32 |
| Immune System | 17 | AKT1\|CASP9\|CDH1\|EGFR\|ERBB3\|MAP2K1\|MDM2\|MTOR\|NFKB1\|NRG1\|PDGFRA\|PIK3R1\|PRKCG\|PRL\|PRLR\|RAB7A\|SRC | REACT:6900 | 2.04E-08 |
| Cell Cycle | 14 | BRCA2\|CCND1\|CCND2\|CCND3\|CDC25A\|CDK4\|CDK6\|CSNK2A1\|DHFR\|HDAC1\|MDM2\|TOP2A\|TUBB\|TYMS | REACT:  115566 | 4.59E-10 |
| Glioma | 13 | AKT1\|CCND1\|CDK4\|CDK6\|EGFR\|IGF1R\|MAP2K1\|MDM2\|MTOR\|PDGFRA\|PIK3CG\|PIK3R1\|PRKCG | KEGG:05214 | 4.41E-21 |
| Focal adhesion | 13 | AKT1\|CCND1\|CCND2\|CCND3\|EGFR\|IGF1R\|MAP2K1\|PDGFRA\|PIK3CG\|PIK3R1\|PRKCG\|RARG\|SRC | KEGG:04510 | 3.69E-14 |
| ErbB signaling pathway | 11 | AKT1\|CCND1\|EGFR\|ERBB3\|MAP2K1\|MTOR\|NRG1\|PIK3CG\|PIK3R1\|PRKCG\|SRC | KEGG:04012 | 2.14E-15 |
| MAPK signaling pathway | 10 | AKT1\|CASP3\|EGFR\|HSPB1\|MAP2K1\|NFKB1\|PDGFRA\|PRKCG\|TGFB1\|TNF | KEGG:04010 | 7.76E-09 |
| p53 signaling pathway | 9 | BAX\|CASP3\|CASP9\|CCND1\|CCND2\|CCND3\|CDK4\|CDK6\|MDM2 | KEGG:04115 | 3.16E-13 |
| Endocytosis | 9 | EGFR\|ERBB3\|IGF1R\|MDM2\|PDGFRA\|RAB7A\|RET\|SRC\|TGFB1 | KEGG:04144 | 1.18E-08 |
| Cytokine-cytokine receptor interaction | 9 | CCL2\|CCL5\|CXCL1\|EGFR\|PDGFRA\|PRL\|PRLR\|TGFB1\|TNF | KEGG:04060 | 9.18E-08 |
| VEGF signaling pathway | 8 | AKT1\|CASP9\|HSPB1\|MAP2K1\|PIK3CG\|PIK3R1\|PRKCG\|SRC | KEGG:04370 | 7.71E-11 |
| Apoptosis | 8 | AKT1\|BAX\|CASP3\|CASP9\|NFKB1\|PIK3CG\|PIK3R1\|TNF | KEGG:04210 | 1.66E-10 |
| Jak-STAT signaling pathway | 8 | AKT1\|CCND1\|CCND2\|CCND3\|PIK3CG\|PIK3R1\|PRL\|PRLR | KEGG:04630 | 1.54E-08 |
| Chemokine signaling pathway | 8 | AKT1\|CCL2\|CCL5\|CXCL1\|MAP2K1\|NFKB1\|PIK3CG\|PIK3R1 | KEGG:04062 | 7.63E-08 |
| Toll-like receptor signaling pathway | 7 | AKT1\|CCL5\|MAP2K1\|NFKB1\|PIK3CG\|PIK3R1\|TNF | KEGG:04620 | 2.56E-08 |
| Steroid hormone biosynthesis | 6 | AKR1C1\|CYP19A1\|CYP1B1\|HSD17B1\|STS\|SULT1E1 | KEGG:00140 | 1.48E-08 |
| Gap junction | 6 | EGFR\|MAP2K1\|PDGFRA\|PRKCG\|SRC\|TUBB | KEGG:04540 | 3.10E-07 |
| Tight junction | 6 | AKT1\|CDK4\|CDK6\|CSNK2A1\|PRKCG\|SRC | KEGG:04530 | 3.09E-06 |
| Natural killer cell mediated cytotoxicity | 6 | CASP3\|MAP2K1\|PIK3CG\|PIK3R1\|PRKCG\|TNF | KEGG:04650 | 3.48E-06 |
| Regulation of actin cytoskeleton | 6 | EGFR\|MAP2K1\|PDGFRA\|PIK3CG\|PIK3R1\|RARG | KEGG:04810 | 4.01E-05 |
| NOD-like receptor signaling pathway | 5 | CCL2\|CCL5\|CXCL1\|NFKB1\|TNF | KEGG:04621 | 8.77E-07 |
| Adherens junction | 5 | CDH1\|CSNK2A1\|EGFR\|IGF1R\|SRC | KEGG:04520 | 2.76E-06 |
| GnRH signaling pathway | 5 | EGFR\|MAP2K1\|MMP2\|MMP9\|SRC | KEGG:04912 | 1.49E-05 |
| Cell-Cell communication | 5 | CDH1\|CDH2\|KRT5\|PIK3R1\|SRC | REACT:111155 | 4.44E-05 |
| Insulin signaling pathway | 5 | AKT1\|MAP2K1\|MTOR\|PIK3CG\|PIK3R1 | KEGG:04910 | 5.81E-05 |
| mTOR signaling pathway | 4 | AKT1\|MTOR\|PIK3CG\|PIK3R1 | KEGG:04150 | 2.02E-05 |

**Table S7. Pathway analysis of breast cancer related targets.**
